# An assessment of uncontrolled human interventions on the contemporary sediment budget and morphological alterations of the Vu Gia Thu Bon River basin, central Vietnam

**DOI:** 10.1016/j.heliyon.2024.e31476

**Published:** 2024-05-28

**Authors:** Binh Quang Nguyen, Sameh A. Kantoush, Doan Van Binh, Tetsuya Sumi

**Affiliations:** a Water Resource Center, Disaster Prevention Research Institute (DPRI), Kyoto University, Kyoto, 611-0011, Japan; b The University of Danang - University of Science and Technology, 54 Nguyen Luong Bang, Danang, Viet Nam; c Master Program in Water Technology, Reuse and Management, Vietnamese German University, Binh Duong, Viet Nam

**Keywords:** Sand mining, Dam construction, Newly proposed empirical formula, Sediment budget, Morphological alterations, Vu Gia Thu Bon River basin

## Abstract

The Vu Gia Thu Bon (VGTB) River basin is critical for regional development and prosperity in water resources. However, human interventions (e.g., dam construction and sand mining) have significantly affected this basin's sediment budget and morphological alterations over recent decades. Such humane actions drive an imbalance in water resources in the basin from upstream to downstream. Therefore, this study investigated spatiotemporal changes in sediment budget and morphology alterations using long-term data and bathymetric surveys; from these data, dams and sand mining contributions were quantified and differentiated. Based on field survey data and interviews, we estimated the sand-mining volume by incorporating reported and a newly proposed empirical formula. The results show that the total riverbed incision volume from 2010 to 2021 was 63.30 Mm^3^, with an incision rate of 0.14 m/yr. The officially reported sand-mining rate was 1.12 Mm^3^/yr, while the newly proposed empirical formula estimated 4.4 Mm^3^/yr. According to the developed empirical formula, the percentage reductions in the sediment budget due to sand mining and upstream dams were 69.7 % and 30.3 %, respectively, according to reports, and 17.8 % and 82.2 %. The statistical method was thus likely too conservative compared to the developed empirical formula. We found that the natural sediment supplies sourced from upstream were insufficient to compensate for the mined bed material. Therefore, our combination of different datasets permitted the assessment of future geomorphological developments within the VGTB River basin under the ongoing sediment deficits. The results of this study provide valuable insights into the impacts of human interventions, specifically sand mining, on the sediment budget, morphological alterations, and riverbed incision. The developed assessment forms the foundation for developing and expanding the region's water/sediment resource management strategies.

## Highlights

• We proposed a novel empirical formula to estimate the annual sand-mining volume.
• The statistical method was thus likely too conservative compared to the developed empirical formula.
• According to the developed empirical formula, the percentage reductions in the sediment budget due to sand mining and upstream dams were 69.7 % and 30.3 %, respectively.
• The natural sediment supply was insufficient to compensate for the loss of extracted bed aggregates.

## 1 Introduction

Sediment transport plays a vital role in morphological alterations and impacts the hydrological cycles of rivers [[1], [2]]. Understanding the sediment budget and morphological alterations in tropical rivers is crucial for ensuring an optimal flow discharge capacity to reduce flood/drought risks and erosion/deposition riverbeds from the mountains to the sea. Empirical evidence from rivers worldwide indicates that dam construction, sand mining, land-use/land cover (LULC) changes, climate change/climate variability, and other human interventions have substantially disturbed the sediment budget [[3], [4], [5], [6]]. Therefore, assessments of the impact of human interventions on sediment loads can provide scientific insights into understanding the hydrological characteristics, sediment budget, and developing river basin management and sustainability strategies [7].

Quantifying sediment loads at the river-basin scale with particular hotspot areas where extensive sand-mining activities, bank erosion, and geomorphologically active channels occur requires detailed spatiotemporal bathymetric data, hydrological and hydrodynamic data, reservoir sedimentation volumes, extracted sediment volumes, bank erosion rates, and channel migration information. These variables affect the total sediment budget calculation due to the variable magnitudes and frequencies of the corresponding drivers and the grain size distribution. Sand-mining activities remove riverbed sediments and create numerous pits [8]. These pits trap bed loads from upstream reaches and prevent them from traveling downstream [9]. Sand mining is also involved with further bathymetric changes such as deepening, narrowing, or widening of river channels and deltas, as well as either thalweg shifts and waterway direction [[10], [11], [12], [13], [14]]. Brunier et al. [15] used bathymetry between 1998 and 2008 and claimed that the average extraction along the entire VMD is 35.5 Mm^3^/year. Gruel et al. [16] indicated that sand mining is conducted on a large scale in the VMD, and the amount of sand extracted in 2018 was 43.6 Mm^3^. Binh et al. [17] found that sand mining is responsible for annual riverbed incisions of 14.8 % in the VMD. Riverine mining also decreased the flood frequency in the Long Xuyen Quadrangle in the VMD by 7.8 % from 2005 to 2015 [8]. In addition, erosion of the river mouth due to sand mining has been recorded in the Pearl River Delta [18], as well as the decline in tributary areas along the Kerala coast [19] and coastal erosion in Tamil Nadu [[20], [21]]. The area of these adverse impacts extends from the local area to larger areas, far from sand mining sites [22]. In addition, reservoirs trap a substantial amount of sediment, thus reducing the sediment supply downstream. In China's Yangtze River, dams (e.g., Three Gorges Dam) have reduced flood flows, increased dry flows, and decreased sediment loads, consequently leading to the degradation of river morphology [[23], [24],^25^]. For instance, the construction of the Sidi Mohamed Ben Abdellah dam on Morocco's Bouregray River in Africa in 1974 significantly lowered the downstream sedimentation rate, from about 3.64 cm/year (1950–1978) to roughly 0.41 cm/year between 1990 and 2017 [26]. Therefore, a full investigation of basin-scale changes can help improve water resource/sediment management and planning.

**Fig. 1:**
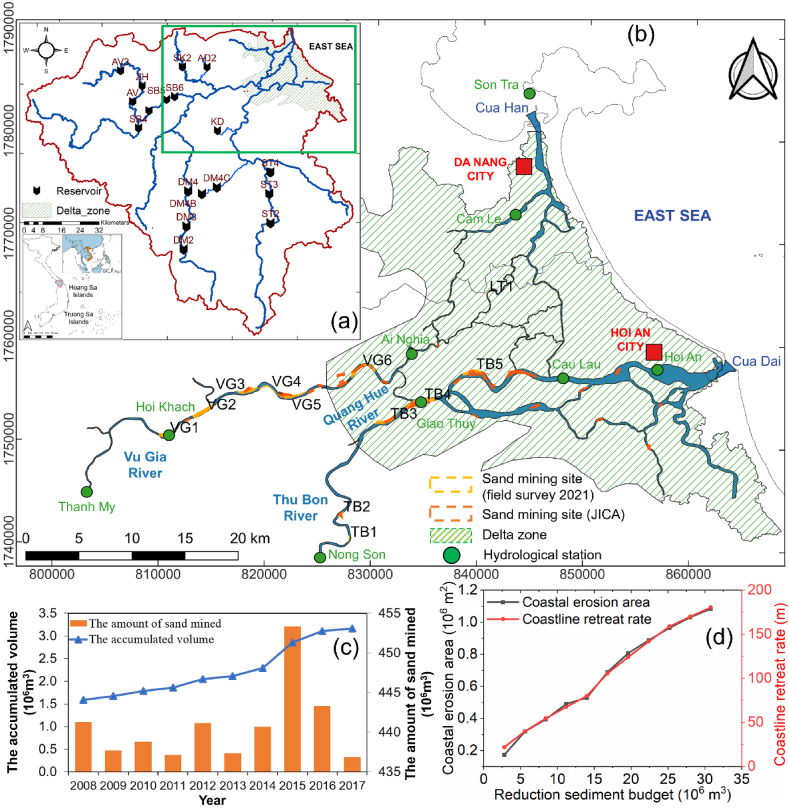
(a) Location of the VGTB River basin, including river systems and dam locations. The full names of the labeled dams are shown in Table A.1. (b) The VGTB River network, hydrological stations, and sand-mining sites on the VGTB River from 2008 to 2017 [32] and in 2021 from a field survey of authors. (c) The amount of sand mined from 2008 to 2017 and the accumulated mined volume from 1990 to 2017. (d) The relationships between the reduction in the sediment budget in the Thu Bon River and the coastal erosion area and between the sediment budget and the coastal retreat rate from 2010 to 2021.

The total water volume of the VGTB River basin accounts for approximately 2.5 % of Vietnam's water and produces approximately 1.5 % of the national GDP. However, the basin is highly impacted by disasters, with damage costs of approximately 7 % of the basin's GDP per year [27]. The VGTB basin is under pressure from human stresses such as LULC change, dam development, and sand mining activities [28]. The intensive development of dams and reservoirs in the last two decades has changed the flow regime and reduced the sediment load of this river, consequently damaging the coastline (Fig. 1b) [[29], [30], [31]]. Ha [31] indicated that the sediment flow from the Thu Bon River was reduced by half compared to the period before the construction of the upstream reservoir. In addition, unsustainable sand mining seriously impacts rivers, deltas, and the coastal zone of the basin [[27], [32]]. Therefore, the basin is experiencing negative impacts from sand mining, and some other impacts are expected to appear similar to those observed in other basins worldwide. However, the sand mining volume appears to be underestimated in previous studies (e.g., [[27], [32]]) because illegal sand mining was not accounted for in the statistics provided by sand miners or local authorities, as highlighted in the Mekong River basin by Binh et al. [17] and Gruel et al. [16]. Moreover, the effect of sand mining on morphological alterations in the VGTB River basin was not fully understood and properly estimated because of data availability.

Hoi An (located in the downstream VGTB River basin) is an ancient city and has been classified by UNESCO as a World Cultural Heritage site (Fig. 1b). However, since 2004, the topic of erosion has been a concern in Hoi An, as erosion threatens to make this old town disappear. The shoreline has retreated, and the erosion area has expanded rapidly. Most resorts and hotels along 7 km of the ancient coastline have been damaged, resulting in economic losses [32]. Even after repairs and hard-measure construction work have been implemented to reduce erosion risk, this problem has occurred continuously, and river basin assessment results are still unclear. Downstream of this area are two large economic regions (Da Nang City and Quang Nam Province) with rapid urbanization and economic development [33] (Fig. 1b). Rapid population growth, coupled with rapid urban development and industrial zones, puts pressure on forest ecosystems, land resources, water/sediment resources, and coastal resources [28]. In addition, securing water and building materials is very important for these regions. These resources are mainly mined from sources within the VGTB River basin [[27], [32]].

Because of a lack of data, previous studies that analyzed the sediment budget in the VGTB River basin did not comprehensively analyze these drivers at the basin or reach scale. Most past studies have concentrated mainly on hydrology under the impact of dam operation and climate change/climate variability [[34], [35], [36], [37]], and geomorphological studies have focused on the estuary [38,39] (Fig. 1b). These studies have research gaps in that they did not assess the sediment budget or morphological alterations from the mountain region to the river outlet, from the upstream to the delta [[40], [41], [42]]. JICA [32] applied the Ashida-Michiue formula to the sand-carrying capacity to calculate the sediment amount transported through cross-sections at various locations. However, the authors only apply it to some typical locations. In addition, this approach does not consider all drivers, including spatiotemporal changes. Uncontrolled sand mining in the downstream VGTB River basin has also significantly reduced the amount of sand entering the sea [32]. Therefore, this study aims to provide useful and invaluable insights into the effects of dams and sand-mining activities on the bathymetry, sediment budget, and morphological alterations in the VGTB River basin.

The specific objectives of this work are to 1) understand the geomorphological conditions of all processes along the rivers and coastlines due to human impacts, 2) separate the impacts of the upstream dam and sand-mining activities on the sediment budget, and 3) link the upstream sediment reduction and increased sand-mining activities to the spatiotemporal changes in the sediment budget and geomorphology. This study's results provide valuable insights into the impacts of human interventions on the sediment budget and morphological alterations. This study's new contribution involves quantifying sand-mining activities and the dam's impact on the sediment budget of the VGTB River basin using the newly proposed empirical formula. This approach can be applied to other basins to clearly understand the impacts of human interventions on the local sediment budget and geomorphology.

## 2 Materials and methodology

### 2.1 study area

The VGTB River basin is located in a tropical monsoon climate zone (Am-Koppen climate classification), encompassing a total area of approximately 10350 km^2^ (Fig. 1a). The study area has a unique topography with mountain ranges running from west to east. The basin's geography is primarily mountainous, with an average slope of 25 %, accounting for 60 % of the entire catchment area, and the slope varies from 0 to 70° [[30], [43]]. The lithology in the basin is mainly sedimentary, igneous, and metamorphic rocks.

The distribution of rainfall is temporally and spatially uneven, varying significantly between seasons and the geographical terrain of the region. From 1980 to 2020, the basin received yearly rainfall varying from 2184 mm to 4188 mm [44]. The region experiences a distinct rainy season (September–December), with peak rainfall from October to December, elevating the flood risk. Flow accounts for approximately 62.5 %–69.2 % of annual flow discharge. Conversely, the prolonged dry season from January to August witnesses minimal rainfall, often leading to water shortages and salinity intrusion. The hilly terrain features and significant rainfall characteristics bring enormous potential for hydropower energy of the basin (Fig. 1a–Table A.1). Up to now, 18 reservoirs have been strategically deployed for hydropower, agricultural production, and water supply purposes in the basin [45]. Out of a total of 18, a significant 12 reservoirs have been successfully constructed in the Vu Gia sub-basin (refer to Fig. 1a). The VGTB River is generally characterized by alternating sand bars on both riverbanks and a large bank full capacity (Fig. 1b). The investigated long section of the VGTB River basin covers various bends with sinuosities ranging from 1.05 to 2.1. VGTB is the watershed for two major rivers: the Vu Gia and Thu Bon Rivers. The distance from the Vu Gia River upstream to the outlet is 204 km. The Thu Bon River originates from a 1500 m high mountainous area and flows into the East Vietnam Sea at the Cua Dai estuary, with a length of 198 km (Fig. 1b). Water is exchanged between the Vu Gia and Thu Bon sub-basins through the Dak Mi 4 hydropower plant (diversion tunnel) and Quang Hue Channel (Fig. 1a and b). Quang Hue is a natural channel located in the middle basin, near the delta apex, that has changed in location over time [28].

The key characteristic is the high sediment load in the main river compared to the tributaries, which is why most sand-mining activities are located in the main river channel (Fig. 1b). Sand mining occurs in instream areas located on floodplains with suitable underlying geology along the VGTB River. The report and field surveys show that extraction methods include dredging boats, suction pumping, and bar skimming, and such activities occur both in daylight and at night. The method type choice depends on the river's characteristics and the ability to implement extraction. In the VGTB River basin, where demand is greatest, monitoring and controlling these activities is difficult. Therefore, the volume of sand extracted from rivers is hard to pinpoint.

### 2.2 methodology

**Fig. 2:**
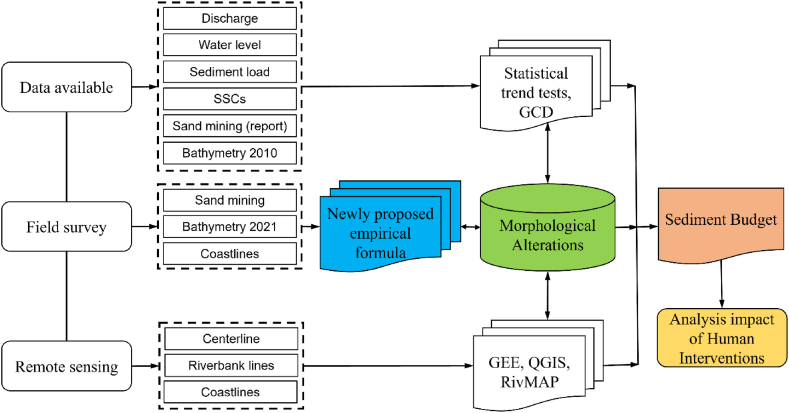
Framework of the current study.

The general flowchart/schematic diagram of this study is shown in Fig. 2. We collected available data and combined these data with field surveys, remote sensing, and analytical methods to comprehensively investigate the effect of human interventions on the sediment budget and morphological alterations.

Erosion and deposition are natural processes in river systems. Erosion in one location is the source of deposition in another location. However, we can determine the increasing or decreasing trends and volumes when we estimate the sediment budget for a river system over a long-term period. Studies have indicated that natural, human interventions or a combination of both can be the causes of riverbed deformation [[46], [47],^48^]. In the VGTB River basin, human interventions including sand mining, and dams are the two dominant drivers of sediment budget calculation [32,45]. Therefore, our methodology for sediment budget estimation is that if the sediment is lost over time from the riverbed, the lost sediment is attributed to both sand mining and dams. Because dams indirectly cause riverbed incisions by trapping the sediment supply, it is difficult to quantify the impact of dams on sediment deficits. First, we quantify the effect of sand mining on riverbed incision volume. The remaining sediment lost from the riverbed is thus attributed to upstream dams. This methodology was well recognized by Binh et al. [[4], [17]].

#### 2.2.1 data collection

The main focus of this paper is assessing human interventions directly in the delta region. In this study, the available continuous daily discharge and suspended sediment concentrations (SSC) data spanned the 1996–2020 period were obtained at two stations located in the upstream region of the VGTB River basin, namely, Thanh My and Nong Son (Fig. 1a and b). These are the only two stations in the basin that monitor discharge and SSC.

The Department of Natural Resources and Environment (DONRE) manages mineral resources in Vietnam. The DONRE manages and licenses the operation of sand mining activities. According to the DONRE of Quang Nam Province, there were approximately 85 sand-mining sites with a total volume of 443 Mm^3^ in the region from 1990 to 2007. We also collected sand-mining data from the Japan International Cooperation Agency (JICA) and Quang Nam's DONRE from 2008 to 2017, with total annual volume ranging from 0.3 to 3.2 Mm^3^/yr. Assessment during the 2011–2017 period shows that the average annual amount of sand mined was approximately 1.12 Mm^3^/yr (Fig. 1c).

#### 2.2.2 field survey

**Fig. 3:**
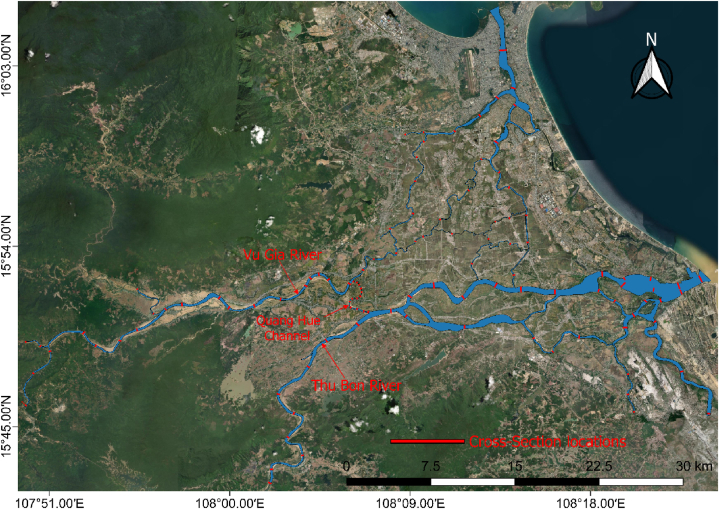
Map of cross-sectional locations surveyed.

The field surveys were conducted along the VGTB River system, with a total length of approximately 240 km. The surveys were performed in March 2021 and April 2022, before the start of the flood period (Fig. 3). The measured extent was from the Thanh My station to the river mouth of the Vu Gia River (Cua Han) and from the Nong Son station to Cua Dai, the river mouth of the Thu Bon River [[28], [49]] (Fig. 1, Fig. 3). Acoustic Doppler Current Profiler (ADCP), Odom Hydrotrac II combined with the Trimble R5 and R8 GPS was used during field surveys [50,51]. Odom Hydrotrac II was used to calibrate the ADCP measurement results. In 2010, 71 cross-sections with an average distance of 1.0–3.2 km were measured at each location. The left and right bank limits are marked at each cross-section to ensure that surveys are conducted at the same route. The floodplain and riverbank of each cross-section were surveyed using Trimble R5 and R8. These machines have mean horizontal and vertical survey errors of ±0.003 m and ±0.005 m, respectively. The sampling time, hydrological conditions, and boat speed were carefully observed to ensure measurement quality during the field surveys. After measuring the underwater and above-water elevations at 71 cross-sections, we processed the raw data to obtain point data in the form of X, Y, and Z coordinates in each cross-section.

Hourly water level data at nine hydrological stations along these rivers were also collected (namely, Thanh My, Hoi Khach, Ai Nghia, Cam Le, Son Tra, Nong Son, Giao Thuy, Cau Lau, and Hoi An) to extract bathymetry data (Fig. 1b). The WinRiver II and the ADCP tool were used to extract raw data [[52], [53]]. From the water level data, we used a distance and time information matrix to interpolate the cross-sections. Finally, we obtained the riverbed elevation at cross-sections by subtracting the water level from the river depth. The bathymetric datasets were converted to the WGS 1984, zone 48 North projection using the Vietnam national datum at Hon Dau.

#### 2.2.3 Remote sensing image and google earth engine platform

We used different analytical methods to understand the roles of climate variation and human intervention impacts on planform adjustments of the VGTB River. The Google Earth Engine (GEE) and River Morphodynamics from Analysis of Planforms (RivMAP) were selected to quantify planform changes at multidecadal time scales (1973–2021). The accuracy level and uncertainty depend on the quality and resolution of the planform mask. Therefore, QGIS software using Python API and integrated GEE was also applied to export the real images and recheck the other analysis results. The results of multispectral, multitemporal satellite imagery analyses provide insight into the dynamics and geomorphic of large rivers. The outputs from GEE were used herein to derive planform statistics and quantify the corresponding changes. In this study, we use the RivMAP toolbox with the single-thread nature of the active channel to analyze the centreline [[54], [55]]. The analysis results have been tidally corrected, particularly in the coastal area where the tide significantly influences the river water level [[56], [57]].

#### 2.2.4 data processing and analysis

a. Statistical trend tests

Data from two stations were used to fully examine the daily streamflow and sediment load changes (Fig. 1b). We identified the data trends by using the nonparametric slope methods of Mann-Kendall and Sen (p = 0.05) to estimate the change rates [[58], [59], [60], [61]].

b. Suspended sediment loads

The daily SSLs were calculated from the daily discharges (Q) and corresponding daily SSC:

(1) $$SSLs=\frac{86400\times SSC\times Q}{10^{6}}$$

SSC, Q, and SSL units are g/m^3^, m^3^/s, and t/day, respectively. The SSC samples were determined following a standard depth-integrated sampling procedure. The samples were taken daily at 7 a.m. using a bottle with a volume of 2 L, preserved, and transported to the laboratory.

c. Sand-mining estimation

Novel approaches must be examined to assess heavily human interventions, such as sand mining for sediment budgeting in tropical rivers. There are various contradictory numbers evaluated by conventional approaches, in which the most common method is from official statistics through mining licenses. However, this method does not assess the amount of illegally mined sand, which may contribute significantly to total sand mining [[16], [17]]. Previous researchers utilized different approaches along the Tien River (Vietnamese Mekong River system) to measure the extracted sand volume, e.g., Jordan et al. [62]. In addition, Hackney et al. [63,64] focused on assessing bank instability associated with sand mining activities and determining the amount of sand using PlanetScope imagery in Cambodia. However, complex natural flow processes can erase traces of sand mining through erosion, deposition, or reworking. A new approach uses remote sensing to quantify sand mining activities and barge traffic, e.g., Gruel et al. [16]. The authors used the correlation of remote sensing and measurement data to quantify sand-mining extraction in the VMD. This method requires many different types of data, including those from surveys that specifically identify boat types, as a basis for boat classification and bathymetry difference maps. Therefore, this method is difficult to apply to data-limited basins. In this study, we estimate the sand-mining volume over the tropical river of the VGTB River basin using a newly proposed empirical formula based on various field survey data and questionnaires. From that, how can we estimate the annual volume of sand mined to help us better understand the human intervention impact on the sediment budget of the VGTB River basin?

Firstly, we mapped the sand mining sites and volume at each site from the government's report. Based on this map, we recounted data from two surveys in March 2021 and April 2022. The sand mining sites are stable and consistent with the report. However, the capacity and volume of sand mining tend to be higher. Also, the volume of sand extracted from rivers is hard to pinpoint. Currently, the government is trying to control the volume of sand mined. However, enforcement has proven difficult due to miners' limited capacity, low investments, and lack of environmental awareness.

Based on field survey data and interviews with people around all sand mining sites, assuming that these sites are stable and the volume of sand mined is consistent annually, the authors propose a formula for estimating the total volume of annually extracted sand. Specifically, we counted the number of machines in operation at each site and interviewed people around each site to assess changes over time and supplement necessary information. We also interviewed the boaters who work daily on the river about the sand mining situation over time and along the river system, and interviewed the owners of barges about the capacity of barges, the average time required to fill a barge, and the working time each day and year. The newly proposed empirical formula is as follows:

(2) $$V_{e}=\frac{\alpha \times C\times n\times T}{t}$$

Where α=0.85(0.8−0.9) refers to the exploitation efficiency factor, obtained by considering the influence of weather, the damage to machines, the transport and break times of machines, and the mining speed between day and night; C = 300 m^3^ (270–330 m^3^) refers to the capacity of each barge; n = 12 (11–13) denotes the total number of machines in operation at each mining site (dredging boats, suction-pumping, and bar-skimming machines), averaged from two field surveys conducted in March 2021 and April 2022; t = 3.5 h (3.2–3.9 h) denotes the average time required to fill a barge; and T = 5040 h (4536–5544 h) is the total annual sand mining duration (8 months during the dry season, from January–August; the average duration over one month was 30 days, and the average duration over one day was 21 h).

d. Bathymetric data analysis

Many bathymetric interpolation methods were evaluated in this study. Interpolation methods are available in the Analyst module (ArcGIS®10.4.1). The universal kriging method achieves the smallest error of all methods from tests based on 2021 data. This interpolation method was then used for the 2010 bathymetric data. The dataset was resampled and clipped to match the study area with a 10-m resolution. The Digital Elevation Models (DEMs) are used to estimate the sediment budget during the period by Geomorphic Change Detection (GCD) [65,66]. The volume change was determined and analyzed from the DEMs.

## 3 Results

### 3.1 Sediment loads and sand mining from the mountains to the sea

**Fig. 4:**
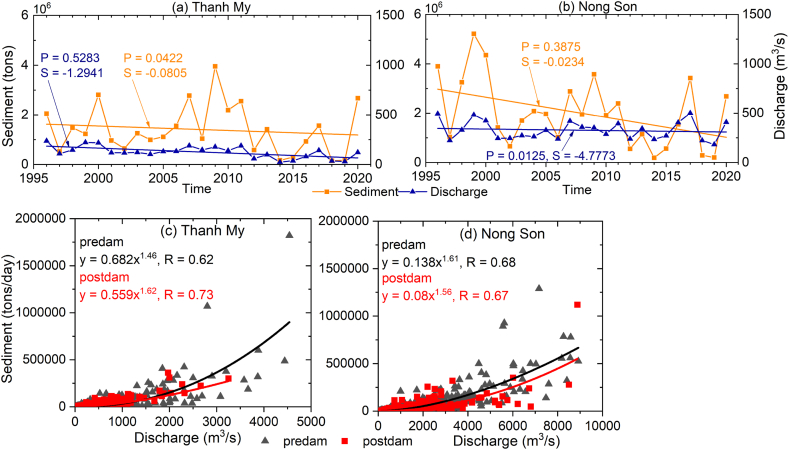
(a), (b) Annual flow discharge and sediment load data recorded at the Thanh My and Nong Son stations from 1996 to 2020. (c), (d) Relationships between the discharge and sediment amounts in the predam and postdam periods.

The annual average flow discharges at Nong Son station slightly decreased (S = 4.7773 m^3^/s/yr), while the sediment load dropped sharply (S = 0.0234 Mt/yr) (Fig. 4b and d). Alternatively, the flow discharge and sediment decreased sharply at Thanh My station (Fig. 4a and c). The sediment load decreased sharply in 2011 when the Dak Mi 4 dam was constructed in the Vu Gia sub-basin, and the Song Tranh 2 dam was constructed in the Thu Bon sub-basin. Therefore, we divided the period into two periods, the predam (1996–2010) and postdam (2011–2020) periods, to examine the effects of upstream dams (Fig. 4). At both stations, the sediment rating curve in the postdam period was lower than that in the predam period. This result indicated sediment trapping by upstream dams (Fig. 4c and d).

**Fig. 5:**
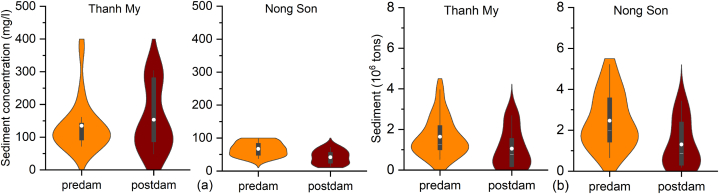
Violin plots representing the kernel densities of the (a) daily suspended sediment concentrations and (b) annual sediment loads at the Thanh My and Nong Son stations in the predam (1996–2010) and postdam (2011–2020) periods.

The violin plots of the mean daily SSC show that the SSC significantly decreased from the predam to postdam periods at Nong Son station (Fig. 5a). The decreasing rate of the SSC was 25 g/m^3^ (from 66.9 g/m^3^ to 41.9 g/m^3^). However, the mean daily SSC increased slightly from 135 g/m^3^ to 153 g/m^3^ at Thanh My station. The data spread was greater in the postdam period than in the predam period. However, the postdam SSC were less variable than those in the predam period. The highest-probability results at the two stations were concentrated in the median values in the predam period (Fig. 5a).

The annual sediment load decreased at the two stations in the postdam period compared to the predam period (Fig. 5b). The mean annual sediment in the entire basin decreased by 1.8 Mt/yr, and those at Thanh My and Nong Son decreased by 0.6 and 1.2 Mt, respectively. The total sediment volume of the entire VGTB River in the postdam period was reduced by 12.5 Mm^3^, with an average reduction of 1.3 Mm^3^ per year (bulk density of 1.4 t/m^3^). These values were in agreement with the JICA report in 2018. After the construction of the dams, the sediment was reduced by 1.0 (Mm^3^/yr) compared to that measured before the construction of the dams [32].

**Fig. 6:**
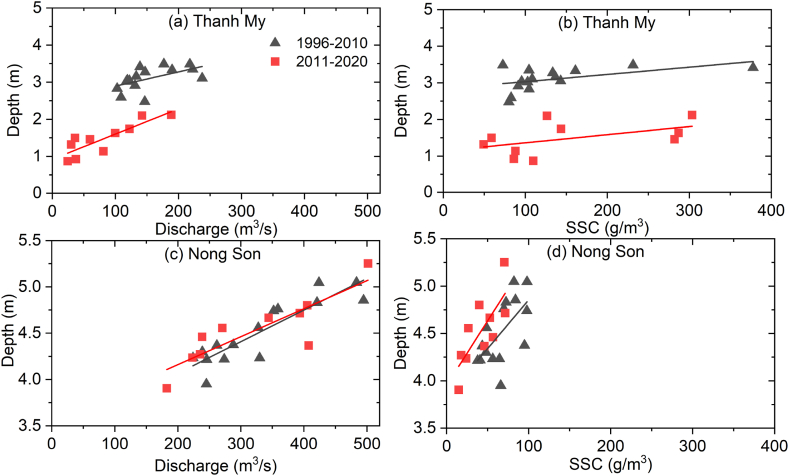
Relationships between the depth of the main channel and the annual moving-average flow discharge and between the channel depth and SSC at Thanh My and Nong Son stations from 1996 to 2020.

The river depth was positively correlated with both the average flow discharge and SSC (Fig. 6). At Thanh My in the Vu Gia River, the annual discharge drastically decreased by 46.7 % (from 154.1 m^3^/s to 82.1 m^3^/s) (Fig. 4, Fig. 6a), while at Nong Son in the Thu Bon River, the annual flow discharge increased in the postdam period (Fig. 4, Fig. 6c). The SSC increased in the 2011–2020 period at Thanh My (Fig. 4, Fig. 6b), and this adjustment was dominant along the transverse direction (Fig. 8c). Alternatively, the SSC decreased at Nong Son (Fig. 4, Fig. 6d). This adjustment was dominant along the vertical direction (Fig. 8d). The differences between the SSC and the sediment transport capacity were significantly reduced at Nong Son, located downstream of multiple dams (Fig. 1, Fig. 6d).

**Fig. 7:**
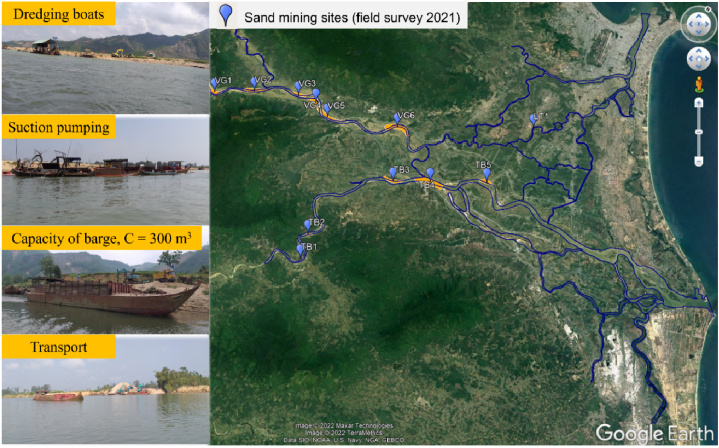
Sand-mining activity in the VGTB River basin (the author took photos during the field surveys).

According to the field surveys, 12 large sand mining sites were identified (seven sites on the Vu Gia River and five on the Thu Bon River) (Fig. 1, Fig. 7). There were three hot-spot sand-mining sites on the Vu Gia River (VG3, VG4, and VG5) and three sites on the Thu Bon River (TB3, TB4, and TB5). Based on the empirical Eq. (2), the authors estimated that the annual sand-mining amount was approximately 4.4 Mm^3^/year, 3.9 times higher than that reported by JICA in 2018 (Fig. 1c).

**Fig. 8:**
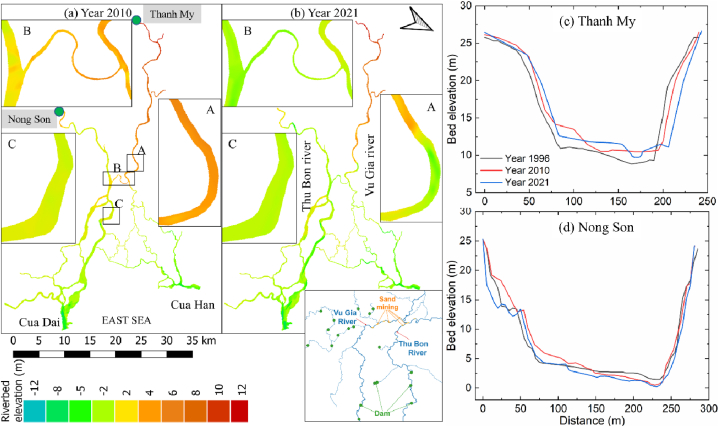
(a) Riverbed elevation in 2010, (b) riverbed elevation in 2021 in 3 subregions: A - the Vu Gia River, B - the Quang Hue Channel, part of the Vu Gia and Thu Bon Rivers, and C - the Thu Bon River, and (c)–(d) riverbed elevations at the Thanh My and Nong Son stations in 1996, 2010, and 2021.

### 3.2 Geomorphological alterations from the mountains to the sea

Detailed comparisons of the bathymetry map across the study period show that the VGTB River system revealed severe riverbed incision, with an incision rate of 0.14 m/yr (Fig. 8a and b). These morphological alterations were concentrated mainly in the middle regions of the Vu Gia River, Thu Bon River, and along the Quang Hue Channel (especially inlet and outlet areas).

**Fig. 9:**
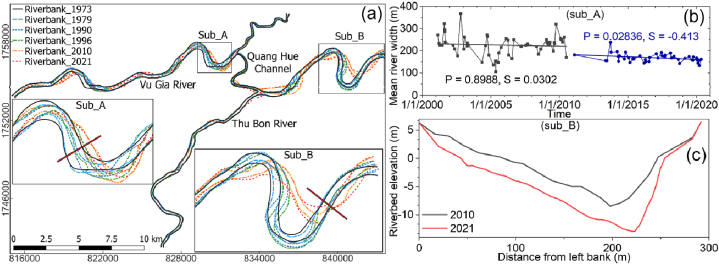
(a) The riverbank of the VGTB River system in 1973, 1979, 1990, 1996, 2010, and 2021. (b) The mean and trend of river width from 2001 to 2020 at sub_A Analytical techniques are inherited from the research results of Yang et al. on the GEE platform. (c) The riverbed elevation in 2010 and 2021 at sub_B.

In the Vu Gia River, riverbed incisions occurred in the middle and upstream reach, and the interesting point is that erosion sites are concentrated mainly in straight river sections (subregions A and B) (Fig. 8a and b). Meanwhile, the erosion points developed strongly downstream reach in the Thu Bon River, and erosion was located in the bend reach (subregions B and C) (Fig. 8a and b). Fig. 8c and d shows that the forms of adjustment to the channel shape at Thanh My station included lateral migration and aggradation, while those at Nong Son station were expansion and incision.(see Fig. 9a).

The length, width, and meandering of the VGTB River system vary greatly over several decades (Fig. 9a and b). The channel width largely increased in the 2001–2010 period and then decreased in the 2011–2020 period due to sand-mining activities and dam developments (Fig. 9b). The river has also narrowed (Fig. 9c).

**Fig. 10:**
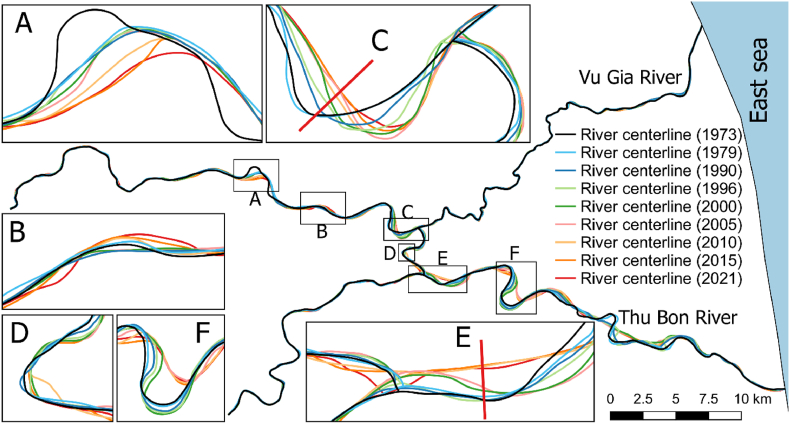
The centrelines of the VGTB River system in 1973, 1979, 1990, 1996, 2000, 2005, 2010, 2015, and 2021.

The river planform changes between 1973 and 2021 along the VGTB River system are shown in Fig. 10. Responses of the tributaries to the mainstream changes were also observed along the VGTB River, especially for the Quang Hue Channel. The centreline locations changed greatly along the curved river and at the inlet and outlet of the Quang Hue Channel. The centreline migrated upstream along the Vu Gia River (subregions A, B, and C) and downstream along the Thu Bon River (subregions E and F) (Fig. 10). The shift distances from 1973 to 2021 at cross-sections C and E were approximately 540 m and 420 m, respectively. The average migration rates at these cross-sections were 11 m and 8.6 m, respectively.

**Fig. 11:**
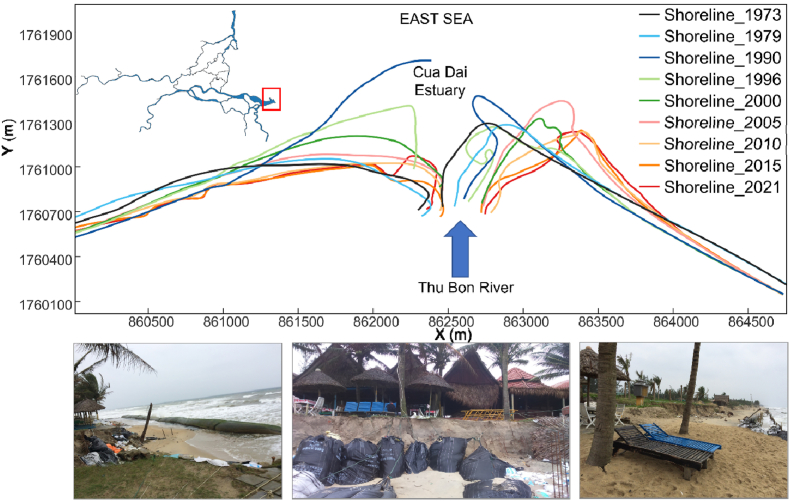
Shoreline variations at the Cua Dai estuary in 1973, 1979, 1990, 1996, 2000, 2005, 2010, 2015, and 2021 as obtained by analysis of remote sensing images. An image of erosion on the left bank in 2016, taken from the author's field survey, is shown. The shoreline data were extracted from historical Landsat-5, Landsat-7, and Landsat-8 imagery. Landsat images courtesy of the USGS were downloaded from the USGS EROS Center.

1Our analysis of exemplary Landsat satellite images revealed massive shoreline erosion within the study area from 1973 to 2021 (Fig. 11). Morphological activity leading to shoreline failure was particularly prominent in the Cua Dai estuary. Fig. 11 shows the clear asymmetry of the shoreline morphological alterations on both sides of the river mouth. The displacement trend of the erosion wedge shifted to the north, starting from the estuary area. The coast of the Cua Dai estuary eroded continuously with a severe grade for a long time (Fig. 1d). The shoreline retreated, and erosion expanded rapidly. The speed of erosion was 10–30 m/year over a length of 7000 m, directly affecting 378 resorts and hotels [32].

### 3.3 Sediment budget changes from the mountains to the sea

**Fig. 12:**
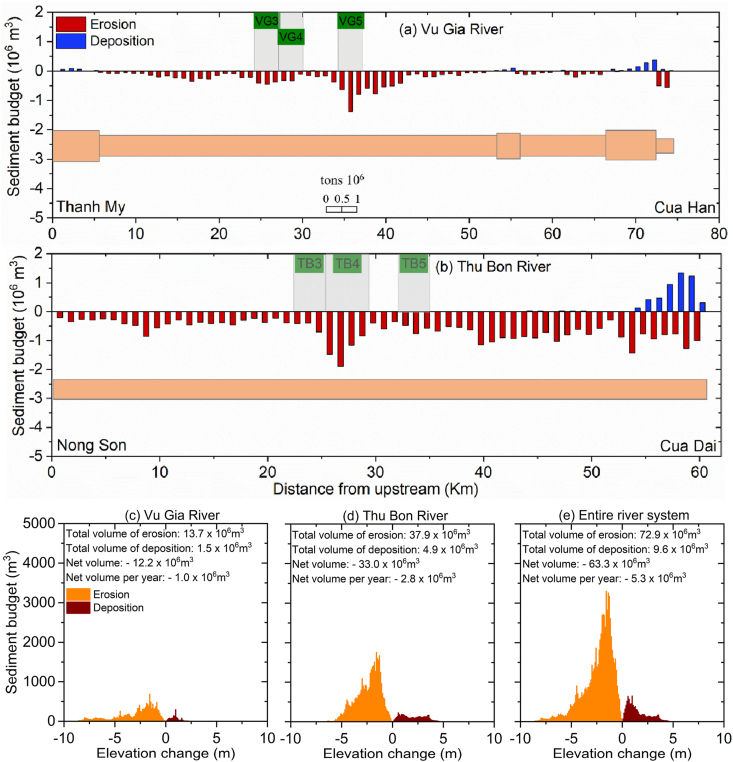
(a)–(b) The sediment budget changes in 2010 and 2021 in the Vu Gia and Thu Bon Rivers. (c)–(e) The sediment budget changes in the Vu Gia River, Thu Bon River, and the entire VGTB River system during 2010–2021.

Analysis of the data shows that the sediment budget has a net deficit across the VGTB River system (Fig. 12). The incision volume during the 2010–2021 period was 63.30 Mm^3^, with an annual average of 5.30 Mm^3^ (Fig. 12e). The riverbeds of the Vu Gia River and Thu Bon River during the 2010–2021 period were incised by 12.2 Mm^3^ (1.0 Mm^3^ per year) and 33.0 Mm^3^ (2.8 Mm^3^ per year), respectively (Fig. 12a, b, 12c, and 12d).

The Vu Gia River's mean incision depth, annual incision volume, and mean incision depth rate were 1.58 m, 1.0 Mm^3^/yr, and 0.13 m/yr, respectively (Fig. 12a and c). Meanwhile, the mean incision depth, annual incision volume, and mean incision depth rate of the Thu Bon River were 1.83 m, 2.8 Mm^3^/yr, and 0.15 m/yr, respectively (Fig. 12b and d). In particular, the mean depth evolution at the sand-mining sites was much larger than that in other locations, typically at VG5 (−7.1 m) on the Vu Gia River and at TB4 (−4.2 m) on the Thu Bon River (Fig. 12a and b).

## 4 Discussion

### 4.1 Effects of upstream dams and sand mining on riverbed incision

Figs. 8b, Figs. 12a and 12b show that riverbed incision in the VGTB River basin occurred at a large scale and was concentrated in sand-mining areas. Therefore, we argue that sand mining is responsible for local- and larger-scale morphological alterations. During the postdam period, the VGTB River was in a net-erosional phase with an incision rate of 0.14 m/yr. The severe riverbed incision within study period was likely due to a sediment reduction (Fig. 4, Fig. 5), not due to changes in the river's transport capacity, as the annual flow discharge of the VGTB River basin is less alterations from 1996 to 2020 (Fig. 4). Therefore, human interventions including sand mining and upstream dams may have been responsible for the riverbed elevation change observed in the VGTB River during the 2010–2021 period.

The incision volume during the 2010–2021 period observed from bathymetric maps was 63.30 Mm^3^ (Fig. 5). According to the report, the total amount of bed material extracted by sand mining was 11.2 Mm^3^ (Fig. 1c), accounting for 17.8 % of the total incision volume (63.30 Mm^3^) in the entire river system. Using the total sand-mining amount estimated from Eq. (2), the total amount of sand exploited was approximately 44.1 Mm^3^, nearly 3.9 times the reported value. This newly estimated sand-mining volume value contributed 69.7 % of the total incision volume. JICA [32] reported that upstream dams and sand mining accounted for 34.5 % and 65.5 % of the annual riverbed incision. Thus, the results estimated from the empirical formula are more reliable and consistent than the reported data. These findings reveal that the sand-mining volume from official reports was underestimated compared to what happens in reality, which is common in other river basins, such as the Mekong River basin [4,16].

The volumes calculated in this study showed discrepancies compared to those reported by the government. This discrepancy is evidence of informal sand-mining activities within the VGTB River basin. Based on observations, the reported values are likely to be significantly lower than the actual values. Dredging activities have increased in recent years, while the statistics are available only up until 2017 (Fig. 1d). Unaccounted-for informal sand mining in official reports is also common in the Mekong region because of the small scale but wide extent of activities by private miners [16]. Sand resources are exploited mainly in the VGTB River basin [[27], [32]] and are difficult to monitor and control. Many construction projects have been implemented under population and economic growth pressure, increasing the demand for aggregates. These aggregates are mainly mined in rivers and are difficult to quantify because most sand mining has been done illegally. The government has tried to stop the illegal extraction of sand from streams and rivers and to improve the management of these forms of mining. However, enforcement has proven difficult due to miners' limited capacity, unclear inspection responsibilities, low investments, and lack of environmental awareness.

Sand mining is responsible for the volume of 44.1 Mm^3^ during the 2010–2021 period, which should be considered a highly conservative estimate within the study area. Despite the uncertainties, it must be highlighted that this is the first accurate sand-mining volume estimated for the tropical river in central Vietnam based on bathymetric maps and by combining empirical formulas and morphological alterations analysis. Previous estimates relied on empirical formulas and assumptions [32]. Finally, our comparison of the effects of sand mining and upstream dams showed that sand mining had the greatest influence on reducing the sediment budget. The percentage reductions due to sand mining and upstream dams obtained from reports were 17.8 % and 82.2 %, respectively. According to the empirical formula, the percentages increased to 69.7 % for sand mining and 30.3 % for upstream dams. These findings highlight that the statistics are likely too conservative. This finding contrasts the VMD, where upstream dams were mainly responsible for annual riverbed incision, and the remaining incision was caused by sand mining (maximum of 14.8 %) [17]. In contrast to the observed extraction volumes from the residual bathymetries and empirical formula, the nonsustainable practices of sand-mining activities within the study area were indicated.

### 4.2 Geomorphological responses to incoming sediment and sand mining

The annual sand-mining volume estimated from the bathymetric maps and empirical formula during the 2010–2021 period indicated that sand mining impacted severe riverbank erosion during this period. The reduced flow discharge and high sediment concentrations caused deposition in the middle and early downstream of the Vu Gia River (Fig. 8a and b). These are major sand-mining sites, and dredging boats have caused riverbank erosion in these areas. Downstream, a portion of the flow discharge is transferred to Thu Bon by the Quang Hue Channel, so erosion in this area is small. According to the monitoring data, the sediment flow and concentration tended to decrease sharply in the Thu Bon River (Fig. 4). Sediment imbalances were observed in the Thu Bon River, especially downstream of the Quang Hue Channel. Therefore, erosion is concentrated downstream (Fig. 8a and b). In recent years, the flow discharge has tended to increase (receiving water from the Vu Gia River), so the phenomenon of "island drift" (migration of small islands towards downstream reaches) has been observed on the river, which has also created serious erosion.

Dams have altered the driven hydrological regime, concurrently leading to a sediment supply shortage for the downstream region. These changes drive changes in the riverbed elevation of the VGTB River (Fig. 8c and d). The cross-sectional river shape adjusts due to the changes in the discharge and sediment load conditions. These changes include changes in the width, depth, and bed level of the river and, as a secondary effect, changes in slope. These changes drive a change in the relationships between the depth of the main channel and the annual moving-average discharge volumes and SSC (Fig. 6). Thanh My station is located upstream and is less affected by dams than is the downstream reach (Fig. 1a). The other sub-basins (without dams, accounting for 39.19 % of the area) contribute greatly to the SSC at Thanh My station. Therefore, the increase in the SSC at Thanh My is mainly due to the increase in the SSC from other sub-basins. The Dak Mi 4 hydropower plant diverts substantial amounts of water from the Vu Gia River to the Thu Bon River, significantly reducing the flow discharge at the Thanh My stations [45]. Collectively, the SSL at Thanh My decreased because of the reduced flow discharge through water diversion by the Dak Mi 4 hydropower plant, although the SSC increased slightly.

The basin has a diversified agriculture, forestry, fishery economy, industry, handicrafts, and services. However, the economic starting point is very low. The economy has been slow to develop for quite some time, mainly via agriculture. The economy has seen a structural change in direction from agriculture to industry and the manufacturing and service sectors [27]. Therefore, the basin is increasingly affected by human interventions. As a result, the tributaries' bedforms also responded to mainstream changes. Changes in water and sediment inputs to downstream reaches may induce changes in planform configuration. Variations in the riverbank, centreline, and width along a river can be used to infer the morphodynamics of the river. Spatially heterogeneous shifts of the river centreline and changes in the trend of the riverbank in the VGTB River indicate the complex morphodynamics of this river (Fig. 9, Fig. 10). The river-centreline changes along the river remained steady until 2010, when the upstream reservoir was impounded.

Morphological alterations are linked directly to bedload transport. The bed load within the study area was quantified indirectly based on the study of Fu et al. [67] and was found to equal 3–15 % of the suspended load. Thus, the annual bedload volume flows in the river basin at the Thanh My and Nong Son stations during the predam period were 0.05–0.25 Mm^3^/yr and 0.07–0.37 Mm^3^/yr, respectively. With these assumptions, the annual bedload transport volumes during the postdam period of 2010–2021 were 0.03–0.16 Mm^3^/yr and 0.04–0.2 Mm^3^/yr, respectively. While the predam bedload kept the river planform stable from 1973 to 2010 (through riverbank lines, Fig. 8a), the reduced bedload in 2010–2021 was partially responsible for riverbank erosion and riverbed elevation in the downstream areas (Fig. 8, Fig. 12b).

Dams reduce the sediment load downstream and, combined with the losses of sediment due to sand mining in the upstream and midstream areas, result in reduced sediment discharge to coastal areas (Fig. 11). Downstream sediment deficits can also adversely affect coastal zones triggering or accentuating beach erosion [[68], [69], [70]]. The reduced sediment supply to the river-mouth area can be related to several factors, such as sand mining along the river or dredging sediment deposition at the rivermouth area. Erosion of the river mouth due to sand mining has been recorded in the Pearl River Delta [18], as well as the decline in tributary areas along the Kerala coast [19] and coastal erosion in Tamil Nadu [[20], [21]]. For the VGTB River basin, these factors have been observed in the Thu Bon basin, and the Cua Dai river mouth area (Hoi An City) (Fig. 11). The relationships between the reduction in the sediment budget in the Thu Bon River and the coastal erosion area and retreat rate from 2010 to 2021 are shown in Fig. 1d. The area has a dynamic river morphology and coastal morphology, with erosion and sedimentation processes affecting the planform and flow resistance of the rivers, the flood risk, and the coastline itself [38,39,[71], [72],^73^]. The mechanisms of shoreline evolution in the Thu Bon River were caused by a combination of human interventions (dams, sand mining, seawalls, etc.) and natural processes (floods, storm waves, tides, etc.) [[74], [75]].

### 4.3 Uncertainty and limitations

A substantial number of uncertainties are inherent in estimating the sand-mining volume in this study. (1) The measurement distance between the cross-sections was large, reducing the quality of the interpolation results, especially at the meandering and tributary sites. (2) The values in the empirical formula are subjective, such as the exploitation efficiency factor and the total number of machines in operation; these values were specific at the surveyed times but may not have been true at other times of the year. The amount of sand exploited varies from 33.7 Mm^3^ to 54.9 Mm^3^, contributing from 53.2 % to 86.8 % of the total incision volume. Therefore, these values must be checked and verified from other field surveys or methods by establishing a more frequent monitoring program to obtain more reliable estimations. However, the sand-mining volumes initially reported from our methods are still valuable for reflecting realistic sand-mining activities in the basin and supporting authorities in planning integrated water-sediment-morphology nexus management by coordinating science-government-community actors.

In addition to upstream dams on the two main rivers, dams are present on the other tributaries of the VGTB River (Fig. 1a). Currently, discharge and sediment volumes are not observed in these tributaries. Thus, we could not fully estimate the changes in the discharge and sediment volumes (suspended and bed loads). We also have not quantified the contribution of SSLs from tributaries and their contribution to the longitudinally variable sediment budget. In addition, the Vu Gia and Thu Bon Rivers are connected in two locations, and the downstream connection is a complex river network (Fig. 1a and b). In this study, we have not assessed sediment contributions from each other and how these contributions affect the constructed sediment budget. To fully understand the effects of the upstream dams and cascade dams on the flow discharge and sediment alterations of the tributaries and the entire river system, it would be necessary to set hydrological and hydro-sediment-morphodynamics models.

## 5 Conclusion

We investigated the impacts of uncontrolled human interventions in the VGTB River basin on the changes in flow discharges and sediment loads over 25 years (1996–2020). We further quantified local- and large-scale morphological alterations in the VGTB River basin from 2010 to 2021 and linked those changes to human interventions. Our findings are summarized.

(1) The annual average flow discharges at Nong Son decreased slightly while the sediment load dropped sharply. We estimated that the annual sediment loads decreased at Thanh My and Nong Son during the predam period by 0.6 and 1.2 Mt/yr, respectively. The mean annual sediment in the Vu Gia and Thu Bon Rivers upstream decreased by 36 % and 47.2 %, respectively, in the postdam period compared to the predam period.
(2) Reducing the downstream sediment loads from upstream large-scale sand mining activities impacts the riverbed incision process. We estimated that the total incision volume during 2010–2021 was 63.30 Mm^3^, with an annual average of 5.30 Mm^3^. The riverbeds of the Vu Gia River and Thu Bon River during the 2010–2021 period were incised by 12.2 Mm^3^ (1.0 Mm^3^ per year) and 33.0 Mm^3^ (2.8 Mm^3^ per year), respectively.
(3) Sand mining had the greatest influence on the intensified riverbed incision in the study area. The percentage reductions due to sand mining and upstream dams obtained from reports were 17.8 % and 82.2 %, respectively. Meanwhile, according to our empirical formula, the percentage decreases were 69.7 % and 30.3 %, respectively. The statistical method was thus likely too conservative compared to the developed empirical formula.
(4) We found that the natural sediment supplies sourced from upstream were insufficient to compensate for the mined bed material. Therefore, our combination of different datasets permitted the assessment of future geomorphological developments within the study area under the ongoing sediment deficits. To ensure sustainable development in the VGTB River basin, the overarching objective must be enforcing regulations, and local sand-mining practices should be monitored and controlled more closely. In addition, field surveys combined with numerical models should also be conducted to provide more insight into the hydrological and morphological processes.

## Data availability statement

The datasets used and analyzed during the current study are available from the corresponding author upon reasonable request.

## CRediT authorship contribution statement

**Binh Quang Nguyen:** Writing – review & editing, Writing – original draft, Visualization, Validation, Software, Resources, Methodology, Investigation, Formal analysis, Data curation, Conceptualization. **Sameh A. Kantoush:** Writing – review & editing, Supervision, Resources, Project administration, Methodology, Investigation, Funding acquisition, Conceptualization. **Doan Van Binh:** Writing – review & editing, Methodology, Investigation, Formal analysis, Conceptualization. **Tetsuya Sumi:** Writing – review & editing, Supervision, Resources, Project administration, Funding acquisition.

## Declaration of competing interest

The authors declare that they have no known competing financial interests or personal relationships that could have appeared to influence the work reported in this paper.

## Footnotes

1 https://earthexplorer.usgs.gov/.
