# Supplementary material for: An assessment of uncontrolled human interventions on the contemporary sediment budget and morphological alterations of the Vu Gia Thu Bon River basin, central Vietnam
Source: Heliyon. 2024 May 28;10(11):e31476. doi: 10.1016/j.heliyon.2024.e31476 (PMC11168294; doi:10.1016/j.heliyon.2024.e31476)
Supplement: Multimedia component 1 [file mmc1.docx]

**Appendix A. Supplementary data**

Table A.1. Reservoir information within the VGTB River basin (Government of Vietnam, 2019).

| Name | Label | Catchment area | Dam high | Normal water level | Total storage | Active storage | Capacity | First year of operation |
| --- | --- | --- | --- | --- | --- | --- | --- | --- |
| Unit | - | km^2^ | m | m | 10^6^ m^3^ | 10^6^ m^3^ | MW | Time |
| A Vuong | AV | 682 | 80 | 380 | 343.55 | 266.48 | 210 | 2008 |
| A Vuong 3 | AV3 | 258.4 | 22.9 | 552.5 | 2.94 | 0.44 | 1.04 | 2016 |
| Song Tranh 2 | ST2 | 1100 | 96 | 175 | 729.2 | 521.1 | 190 | 2011 |
| Song Tranh 3 | ST3 | 1450 | 36.5 | 71.5 | 34.1 | 3.1 | 62 | 2013 |
| Song Tranh 4 | ST4 | 1610 | 25 | 46.5 | 24.81 | 3.32 | 48 | 2020 |
| Dak Mi 2 | DM2 | 445 | 30 | 630 | 1.611 | 0.692 | 98 | 2019 |
| Dak Mi 3 | DM3 | 612 | 30 | 359 | 5 | 2.304 | 63 | 2017 |
| Dak Mi 4 | DM4 | 1125 | 90 | 258 | 312.38 | 158.26 | 148 | 2011 |
| Dak Mi 4B | DM4B | 29 | 23.5 | 105.3 | 0.688 | 0.066 | 42 | 2012 |
| Dak Mi 4C | DM4C | 82.6 | 11.5 | 67.2 | 2.67 | 0.52 | 18 | 2012 |
| Song Bung 4 | SB4 | 1448 | 114 | 222.5 | 510.8 | 233.99 | 156 | 2015 |
| Song Bung 4A | SB4A | 2276 | 46 | 97.4 | 10.6 | 1.58 | 49 | 2012 |
| Song Bung 5 | SB5 | 2369 | 41.5 | 60 | 20.27 | 2.45 | 57 | 2013 |
| Song Bung 6 | SB6 | 2386 | 39.2 | 31.8 | 3.29 | 0 | 29 | 2012 |
| Za Hung | ZH | 537 | 25 | 450 | 1.12 | 0.74 | 30 | 2009 |
| Khe Dien | KD | 72 | 41 | 206.94 | 50.98 | 50.35 | 9 | 2007 |
| Song Con 2 | SK2 | 81 | 48 | 340 | 29.19 | 25.41 | 3 | 2009 |
| An Diem 2 | AD2 | 169.8 | 24.5 | 348.5 | 0.28 | 0.19 | 15.6 | 2010 |
